# A Retrospective Analysis of Three Antiviral Regimens of Peramivir in the Treatment of Severe Influenza A with Primary Viral Pneumonia

**DOI:** 10.1155/2019/3859230

**Published:** 2019-04-30

**Authors:** Jin-na Wang, Xu Wang, Shu-le Yu, Yue-hui Ding, Meng-lei Wang, Hong-dou Chen

**Affiliations:** Department of Pharmacy, The Affiliated Suqian Hospital of Xuzhou Medical University, Suqian, Jiangsu 223800, China

## Abstract

**Objective:**

To evaluate the difference of clinical efficacy of peramivir alone and peramivir combined with immunomodulators (either ribonucleic acid or thymopetidum) in the treatment of severe influenza A with primary viral pneumonia.

**Methods:**

A retrospective analysis was applied to 45 patients who were diagnosed with severe influenza A with primary viral pneumonia in our hospital from December 2017 to March 2018. The cases were divided into three groups: the peramivir group, the peramivir combined with ribonucleic acid group, and the peramivir combined with thymopetidum group.

**Results:**

The duration of viral nucleic acid positivity in the peramivir group, the peramivir combined with ribonucleic acid group, and the peramivir combined with thymopetidum group was 6.13 ± 2.06, 6.53 ± 2.72, and 6.10 ± 1.37 days, respectively. The remission time of the clinical symptoms of the peramivir group, the peramivir combined with ribonucleic acid group, and the peramivir combined with thymopetidum group was 8.06 ± 2.73, 7.94 ± 2.89, and 7.67 ± 1.58 days, respectively. Comparisons between the peramivir group and the peramivir combined with ribonucleic acid group or the peramivir combined with thymopetidum group revealed no significant differences in the duration of virus nucleic acid positivity, remission time of clinical symptoms, time to fever alleviation, and time to cough alleviation.

**Conclusions:**

There is no observed benefit in the addition of ribonucleic acid or thymopetidum when peramivir sodium chloride injection is used in the treatment of severe influenza A with primary viral pneumonia. This trial is registered with ChiCTR1800019417.

## 1. Introduction

Influenza is an acute respiratory disease caused by the influenza virus. Due to its strong infectivity and rapid transmission, influenza has broken out in the world for many times which not only seriously affected the health of people in various countries but also caused immeasurable economic losses [1]. As we know, influenza is mostly self-limited and is usually accompanied by fever, shivering, headache, myalgia, and so on. However, some people such as the elderly, young children, and pregnant and lying-in women can easily develop into severe cases or even deaths because these populations are prone to complications including pneumonia, nervous system injury, and cardiac damage [2–4].

There are currently two categories of anti-influenza drugs. Category I are M2 ion channel blockers, and the main representative drugs are amantadine and rimantadine. These drugs are only effective against influenza A. In addition, because of the high variability of influenza viruses, the resistance to M2 ion channel blockers has been high. Therefore, this class of drugs is not recommended for treating influenza [5]. The second class of anti-influenza drugs is the neuraminidase (NA) inhibitors, such as oseltamivir, zanamivir, and peramivir, which are recommended as the main drugs. Oseltamivir is an oral preparation that cannot be used in critically ill patients and infants under the age of one. Zanamivir is an inhalant, and inhalants are not recommended for severe influenza patients and influenza patients with complications according to the Influenza Diagnosis and Treatment Program (2018 edition, China). The introduction of peramivir sodium chloride injection in China in 2013 has brought good news to patients with severe and critical influenza [6–8].

At present, the research on peramivir in the treatment of influenza is mainly concentrated in patients with simple infection [9, 10]. There are studies in special groups and patients with other diseases (e.g., in patients with diabetes or respiratory diseases or using immunosuppressive agents) [11–15], but few studies have reported the clinical efficacy of peramivir in severe cases [16]. The Influenza Diagnosis and Treatment Program (2018 edition, China) also noted that the current clinical data on peramivir for severe influenza cases were limited. In addition, the outbreak of influenza in northern and southern China since the autumn of 2017 has led to a large number of severe cases and even deaths. Most of these severe cases were complicated with primary viral pneumonia, and there was also a number of patients with a combination of secondary bacterial pneumonia or mixed pneumonia. Therefore, this study focuses on patients with severe influenza A complicated with primary viral pneumonia in our hospital.

In recent years, immunomodulators have been widely used in the treatment of many diseases. The most widely used immunomodulators include ribonucleic acid for injection II and thymopetidum for injection, both of which are used as adjuvant therapeutic drugs for tumors and diseases caused by immunodeficiency [17, 18]. When we study influenza cases in our hospital, we find that peramivir is usually combined with ribonucleic acid or thymopetidum in the treatment of influenza. It may be that clinicians believe immunomodulators can help alleviate influenza symptoms by improving immunity of patients. This finding has attracted our attention, so the purpose of this study is to determine whether it is meaningful in the addition of ribonucleic acid or thymopetidum when peramivir sodium chloride injection is used in the treatment of severe influenza A with primary viral pneumonia.

## 2. Patients and Methods

### 2.1. Diagnostic Criteria

The diagnostic criteria were based on the Influenza Diagnosis and Treatment Program (2018 edition, China). All enrolled patients were diagnosed as severe influenza A with primary viral pneumonia with the following three points. First, the pharyngeal swabs of the patients were tested with reverse transcription polymerase chain reaction (RT-PCR) influenza virus nucleic acid test kits, and the results showed that the patients were positive for influenza A. Second, the patients were diagnosed as primary viral pneumonia (excluding secondary bacterial pneumonia and mixed pneumonia) by clinicians through CRP values (CRP<20 mg/L), bacterial culture (negative results of bacterial culture), and clinical symptoms. Third, one of the following types of cases was considered a severe case: (1) persistent high fever for more than 3 days accompanied by severe cough, sputum, bloody sputum or chest pain; (2) rapid breathing, dyspnea, or cyanosis lips; (3) mental changes that could include slow reactions, sleepiness, restlessness, or convulsions; (4) severe vomiting, diarrhea, or dehydration; (5) combined with pneumonia; (6) significant aggravation of previous underlying diseases. All patients in our study were combined with pneumonia, so they were diagnosed as severe cases.

### 2.2. Patients and Groups

Forty-five hospitalized patients with severe influenza A complicated with primary viral pneumonia admitted to our hospital from December 2017 to March 2018 were selected according to “The Influenza Diagnosis and Treatment Plan” issued by China in 2018. The inclusion criteria were as follows: (1) diagnosed as severe influenza A with primary viral pneumonia and (2) more than 18 years old, regardless of sex. The exclusion criteria were as follows: (1) vaccinated for influenza within the past six months (2) taking M2 channel blockers (e.g., amantadine and rimantadine) and neuraminidase inhibitors (oseltamivir, zanamivir, etc.) within the past month. All patients were divided into three groups: group A (peramivir group), group B (peramivir combined with ribonucleic acid group), and group C (peramivir combined with thymopetidum group).

### 2.3. Therapeutic Method

According to treatment plan of clinicians, patients were divided into three groups. Group A patients were given peramivir sodium chloride injection (Ranbaxy, Guangzhou China). Group B patients received peramivir sodium chloride injection (the same dosage as group A) and ribonucleic acid for injection II (diluted with 5% glucose injection; Jilin Ao Dong Pharmaceutical Co.). Group C patients were treated with peramivir sodium chloride injection (the same dosage as group A) and thymopetidum for injection (diluted with 5% glucose injection; Xi'an Diesel Biological Pharmaceutical Co.).

Peramivir, 300 mg, was intravenously injected once a day. Ribonucleic acid, 100 mg, was intravenously injected once a day. Thymopetidum, 80 mg, was intravenously injected once a day.

### 2.4. Evaluation Indicator

To evaluate the efficacy of peramivir alone and peramivir combined with other drugs (either ribonucleic acid or thymopetidum) in the treatment of severe influenza A with primary viral pneumonia, the duration of virus nucleic acid positivity and remission time of clinical symptoms were selected as the main evaluation indicators. The patient's temperature, respiratory symptoms (such as cough, sputum, sore throat, and nasal congestion), and systemic symptoms (such as muscle pain, chills, and fatigue) were recorded three times a day. The throat swab of patients was collected every two days, and test results were also recorded. The duration of virus nucleic acid positivity referred to the influenza virus nucleic acid going from positive to negative and being maintained for 24 hours or more. The remission time of the clinical symptoms was defined as the disappearance of all influenza symptoms or the presence of only mild influenza symptoms and maintenance of that condition for at least 24 hours. In addition, influenza can cause fever, cough, chills, fatigue, muscle soreness, and other clinical symptoms, so the remission time of these influenza symptoms also reflected the efficacy of these drugs. As shown in Table 1, most patients had influenza accompanied by the two symptoms of fever and cough. Therefore, the remission time of fever symptoms and the remission time of cough symptoms were selected as two secondary indexes to evaluate the efficacy of peramivir alone and peramivir combined with other drugs (either ribonucleic acid or thymopetidum) in the treatment of severe influenza A with primary viral pneumonia. Fever relief occurred when the body temperature dropped to 37.5 degrees and maintained for at least 24 hours. Cough relief referred to no cough or mild cough for at least 24 hours.

### 2.5. Statistical Analysis

Origin 8.0 software was used to process the data. The measurement data were expressed as the mean ± SD, and the numeration data were expressed as the number of cases and %. The *χ*^2^ test was used for group comparisons, and the difference was considered statistically significant when *P* < 0.05.

## 3. Results

### 3.1. General Characteristics

Forty-five hospitalized patients with severe influenza A complicated with primary viral pneumonia admitted to our hospital from December 2017 to March 2018 were included in this study and divided into three groups (A, B, and C).  Table 2 shows the general characteristics of the three groups of patients. The statistical analysis showed that there were no significant differences in these characteristics between the three groups.

### 3.2. Clinical Efficacy

**Figure 1:**
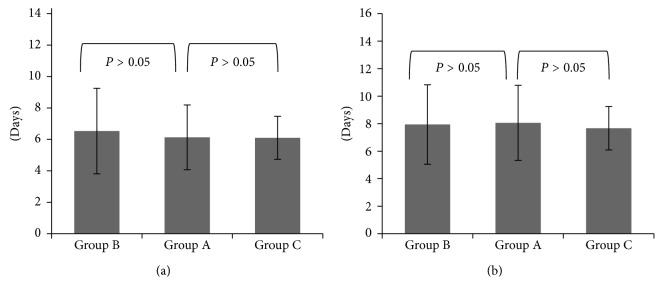
Duration of virus nucleic acid positivity (a) and remission time of clinical symptoms (b) in groups A, B, and C.

As shown in Table 3, the duration of viral nucleic acid positivity in the A, B, and C groups was 6.13 ± 2.06, 6.53 ± 2.72, and 6.10 ± 1.37 days, respectively. There were no significant differences between groups A and B or between groups A and C. In addition, the remission time of the clinical symptoms of the A, B, and C groups was 8.06 ± 2.73, 7.94 ± 2.89, and 7.67 ± 1.58 days, respectively. Compared with group A, the remission time of clinical symptoms in groups B and C was relatively shortened. However, little difference was observed between groups A and B or between groups A and C (Figure 1). The time to fever alleviation in the B and C groups was shorter than that in the A group, but no obvious differences were shown between group A and the other two groups. Lastly, when comparing the time to cough alleviation in group A with that of the other two groups, significant differences were also not detected.

## 4. Discussion

### 4.1. Findings of the Study

In recent years, more and more scholars have paid attention to the topic of peramivir combining with other drugs (such as traditional Chinese medicine injections, Chinese medicine granules, and favipiravir [19]) in the treatment of influenza. Duan reported that peramivir combined with traditional Chinese medicine Qingre recipe has a definite effect on mild influenza and can shorten the remission time of influenza symptoms, which is worthy of promotion in clinic [20]. Li et al. reported that the combination of baicalin and peramivir exhibited a higher efficacy against influenza H1N1 virus infection than they were used alone. The combination of the two agents can not only reduce the dosage of peramivir but also reduce its side effects and drug resistance. The results suggested a potential clinical value of the combination of the two agents [21]. However, there is no report on peramivir combined with immunomodulator in the treatment of influenza at home and abroad. The aim of this study was to determine whether it is necessary to combine one immunomodulator (either ribonucleic acid or thymopetidum) with peramivir in the treatment of severe influenza A with primary viral pneumonia. From the results of the study, we can see that the three treatment regimens can alleviate the symptoms of influenza in all patients and turn the influenza virus negative. However, there was no significant difference in the four evaluation indicators (the duration of viral nucleic acid positivity, the remission time of the clinical symptoms, the time to fever alleviation, and the time to cough alleviation) between the peramivir group and the peramivir combined with ribonucleic acid group or the peramivir combined with thymopetidum group (*P* > 0.05). The results showed that no significant results were observed in the addition of ribonucleic acid or thymopetidum when peramivir sodium chloride injection is used in the treatment of severe influenza A with primary viral pneumonia, which suggests little potential clinical value of the combination of peramivir and one immunomodulator (either ribonucleic acid or thymopetidum). It may be that immunomodulators cannot relieve the symptoms of fever and cough caused by influenza like baicalin or traditional Chinese medicine Qingre recipe. In addition, another reason could be that the immunomodulators made in China cannot improve the immunity of patients in a short time, which cannot help alleviate influenza symptoms. This discovery of this study not only reduces medical expenses and improve medication compliance of patients but also improves the quality of medical services.

### 4.2. Limitations of the Study

The results (Table 3) showed that the average duration of viral nucleic acid positivity and the average remission time of clinical symptoms in the peramivir group were 6.13 and 8.06 days. These durations were significantly shorter than those reported by Chinese researchers Zhan et al. They reported the average duration of viral nucleic acid positivity and the average remission time of clinical symptoms for severe influenza A patients were 11.7 days and 13.21 days (all severe influenza A patients received oseltamivir phosphate capsules) [22]. Although the results show that peramivir can alleviate the clinical symptoms of patients and turn influenza virus negative. And, compared with the existing studies, peramivir has achieved better clinical results than oseltamivir for patients with severe influenza A. However, the absence of a control group (placebo or another neuraminidase inhibitor group) in our study does not confirm the efficacy of peramivir in the treatment of severe influenza A with primary viral pneumonia. Besides, another limitation of the study should also be pointed out. The throat swabs were taken every 2 days, so patients clearing the virus within 48 hours were all considered to have cleared the virus simultaneously. Thus, the estimation for the time to virus clearance lacks precision. In addition, there are some other shortcomings in this study, such as small sample size and lack of multicenter experiments. The reason is that our hospital is small, so we could not collect a large number of samples and we have no funds for multicenter experiments. In addition, the influenza subtype was not identified in this study because of limited experimental conditions. At present, our team is conducting a prospective randomized controlled study equipped with advanced medical equipment to identify influenza subtypes. In addition, due to cooperation with many medical institutions, this study can obtain a large number of samples. It is hoped that more meaningful data can be obtained so as to provide basis for clinical medication.

## 5. Conclusion

According to the results of this study, there is no observed benefit in the addition of ribonucleic acid or thymopetidum when peramivir sodium chloride injection is used in the treatment of severe influenza A with primary viral pneumonia. So, clinicians may consider not to use immunomodulators (either ribonucleic acid or thymopetidum) in combination with peramivir in the treatment of severe influenza A with primary viral pneumonia.

## Figures and Tables

**Table 1:** Comparison of influenza symptoms in the three groups.

Symptom	Group A (*n*=16)		Group B (*n*=19)		Group C (*n*=10)	
	Number	%	Number	%	Number	%
Fever	16	100	19	100	10	100
Cough	16	100	19	100	9	90
Expectoration	13	81.25	13	68.42	3	30
Chilly	3	18.75	10	52.63	7	70
Weak	5	31.25	5	26.32	1	10
Muscle soreness	1	6.25	8	42.11	1	10
Headache	4	25.00	1	5.26	4	40
Sore throat	2	12.50	6	31.58	1	10
Chest tightness	3	18.75	2	10.53	1	10
Nasal congestion	1	6.25	4	21.05	1	10

**Table 2:** General characteristics of the study patients.

Item		Group A (*n*=16)	Group B (*n*=19)	Group C (*n*=10)
Age, years	Mean ± SD	45.80 ± 15.71	44.38 ± 15.01	46.60 ± 15.72
	Range	21–68	22–70	27–67
Sex	Male/female	8/8	8/11	4/6
	%	50/50	42.11/57.89	40/60
Influenza A	Number	16	19	10
Primary pneumonia	Number	16	19	10
Body temperature at admission, °C	Mean ± SD	38.81 ± 0.50	38.49 ± 0.77	38.50 ± 0.91
	Range	38.00–39.70	38.10–39.50	38.00–40.00
Time from onset to administration of drugs (>48 h)	Number	16	19	10

**Table 3:** Clinical efficacy of three groups.

Item	Group A (*n*=16)	Group B (*n*=19)	*P* value	Group A (*n*=16)	Group C (*n*=19)	*P* value
	Mean ± SD	Mean ± SD		Mean ± SD	Mean ± SD	
Duration of virus nucleic acid positivity, days	6.13 ± 2.06	6.53 ± 2.72	0.19	6.13 ± 2.06	6.10 ± 1.37	0.65
Remission time of clinical symptoms, days	8.06 ± 2.73	7.94 ± 2.89	0.47	8.06 ± 2.73	7.67 ± 1.58	0.80
Time to fever alleviation, hours	28.89 ± 16.37	26.93 ± 12.04	0.74	28.89 ± 16.37	27.20 ± 8.87	0.96
Time to cough alleviation, hours	72.00 ± 31.45	69.43 ± 34.99	0.90	72.00 ± 31.45	70.00 ± 42.76	0.99

## Data Availability

The data used to support the findings of this study are available from the corresponding author upon request.
